# Are socio-demographic and economic characteristics good predictors of misinformation during an epidemic?

**DOI:** 10.1371/journal.pgph.0000279

**Published:** 2022-03-16

**Authors:** Elisa M. Maffioli, Robert Gonzalez

**Affiliations:** 1 Health Management and Policy, University of Michigan School of Public Health, Ann Arbor, MI, United States of America; 2 School of Economics, Georgia Institute of Technology, Atlanta, GA, United States of America; Southern Cross University, AUSTRALIA

## Abstract

We combine data on beliefs about the origin of the 2014 Ebola outbreak with two supervised machine learning methods to predict who is more likely to be misinformed. Contrary to popular beliefs, we uncover that, socio-demographic and economic indicators play a minor role in predicting those who are misinformed: misinformed individuals are not any poorer, older, less educated, more economically distressed, more rural, or ethnically different than individuals who are informed. However, they are more likely to report high levels of distrust, especially towards governmental institutions. By distinguishing between types of beliefs, distrust in the central government is the primary predictor of individuals assigning a political origin to the epidemic, while Muslim religion is the most important predictor of whether the individual assigns a supernatural origin. Instead, educational level has a markedly higher importance for ethnic beliefs. Taken together, the results highlight that government trust might play the most important role in reducing misinformation during epidemics.

## Introduction

Health misinformation is considered a major threat to global public health [1], and can exacerbate infectious disease outbreaks. Curbing the spread of infectious diseases requires effective information dissemination strategies [2]. A key objective of these strategies is to mitigate the spread of misinformation, especially during outbreaks of novel diseases such as the Ebola epidemics in West Africa and the Democratic Republic of Congo, and the current COVID-19 pandemic, where much was unknown about the pathogens and treatments were not yet available [3, 4].

The degree of misinformation during epidemics is significant [5–7]. For example, when the 2014 West Africa Ebola epidemic unfolded, denials, conspiracy theories, and false rumors were common [8] and disrupted public health interventions [9–15]. A similar pattern played out during COVID-19 [16–19]. More generally, misinformation is common for other health-related issues such as vaccinations [20–22], nutrition, cancer, fluoridation of water and smoking [3, 8].

While a growing body of research has focused on quantifying the prevalence of misinformation [23], how it spreads [24], its effects on public health [25], and the impacts of interventions to address it [3], very little is known about *who* are the misinformed, i.e., the individuals prone to believe false information. There is a paucity of studies quantitatively investigating the factors associated to misinformation. While age has been primarily associated to sharing fake news, evidence is mixed [26, 27], limited to developed countries’ population and social media, and not taking into account the whole possible set of socio-demographic, economic and political predictors Altruism [28, 29] and self-promotion and entertainment [30] have been also described as motives for sharing fake news. However, this research mainly focused on motivational or personality factors in the technology literature. In Liberia, our country of interest, high levels of distrust in the government existed during the Ebola epidemic [31, 32] and created significant barriers to controlling the disease [31, 33]. Similarly, increased trust was associated with higher utilization of care during the Ebola epidemics in Liberia [31, 34, 35] and the DRC [4]. Yet, there is no quantitative evidence demonstrating whether, among others, trust in the government is a predictor of health misinformation.

We aim at filling this gap in the literature, by studying the characteristics of misinformed individuals during the 2014 Ebola outbreak in Liberia. By conducting a novel individual-level survey shortly after the end of the epidemic, we recorded respondents’ beliefs about the origin of the outbreak: We asked about 2,200 respondents across the country who they thought was responsible for bringing Ebola to Liberia when the virus first appeared. We use these self-reported beliefs to classify individuals as “informed” when they exclusively reported that either traders, people from Sierra Leone or Guinea were responsible, since the epidemic originated at the border with these countries. We classified individuals as “misinformed” if they reported other institutions, including ethnic groups (Fula, Mandingo, Kissi), foreign people (white, UNMIL, foreign Non-Governmental Organizations (NGOs)), the government or others (God, witchcraft). In our data 44% of respondents were informed, while 30% reported that other institutions were responsible for the epidemic, including the government (16%), God (4.2%) or others (6.4%). We combine conventional econometric and machine learning methods with data on economic, socio-demographic, information access, and attitudes indicators to create a profile of misinformed individuals. We also explore their profile by type of beliefs, i.e., whether the epidemic had a political, ethnic or supernatural origin.

## Methods

### Setting

With a population of about 5 million people, Liberia is among the poorest countries in the world with a GDP per capita of $673 and ranking 175 out of 189 countries in terms of Human Development Index [36]. Like many other African countries, Liberia is still characterized by poor health infrastructure and mistrust in state institutions. As such, Liberia is one of the most vulnerable countries in the world to infectious diseases.

From 1989 to 2003 the country experienced two civil wars, shattering the strained relationship between Liberian citizens and their government. When the second war ended in 2003, few Liberian citizens trusted the government for protection or to settle disputes [37]. A decade later another threat to trust came in the form of the Ebola epidemic which hit the country in March 2014. Liberia was the among the three countries most affected by the Ebola epidemic, recording 10,675 confirmed, probable, or suspected cases and 4,809 deaths–the highest number in West Africa [38].

### Data

We use survey data collected between October 2015 and June 2016 on 2,265 individuals across Liberia. Respondents were selected and screened through Random-Digit Dialing (RDD) and a short Interactive Voice Response (IVR) survey, and they were then interviewed by a local NGO through Computer-Assisted Telephone Interviewing (CATI) [39]. The survey tool collected data on: (1) socio-demographic and (2) economic characteristics; (3) access to information; (4) political outcomes, including level of trust in governmental and nongovernmental institutions, perceived corruption, and past voting behavior; (5) Ebola- related exposure questions, including the experience and perceptions around the government’s response; (6) Ebola-related individual perceptions: we asked respondents who they thought was responsible for bringing Ebola to Liberia, both at the beginning when the virus first appeared, and at the time of the interview when Ebola was over. Individuals were not read any options out loud, but the enumerators were instructed to select all categories that applied. In addition to classifying individuals as “informed” or “misinformed” (see main text), 26% of the respondents answered they did not know the origin of the epidemic. Even though we use the “uninformed” definition for convenience of notation, responding “Don’t know” can imply that the respondent may not want to answer the question. Thus, our analysis compares misinformed to informed individuals, excluding those uninformed.

In addition, we combined proprietary data from the Liberia Telecommunications Authority (LTA) on the location of cell phone towers in the year 2013, with the most precise global-scale elevation data available, the 30-meter resolution ALOS Global Digital Surface Model, to estimate an Irregular Terrain Model (ITM) [40] and assess the strength of cell phone coverage. We used this measure of signal strength, defined between -50 and -140 decibel-milliwatts (dBm), to create a dichotomous variable of cell phone coverage. Details on the model and its output are in [41].

Finally, we obtained other data from the Liberia Institute of Statistics and Geo-Information Services (LISGIS), such as Global Positioning System (GPS) coordinates of each village and the 2008 National Population and Housing Census.

### Sample

65% of respondents are male, with more than primary education (67%), wealthier and living in urban areas (70%). The majority of individuals were self- employed (43%) or unemployed (25%) at the time of the interview, and in fact 44% of them reported they worked less or lost their job since the end of 2013. 80% had access to radio, 23% to television, and 69% lives in cellphone coverage area, with the majority (66%) owning a cellphone. Most of respondents trusted the radio and health officials (76%) as sources of information.

On a scale from 0 to 10 (from no to high trust), respondents trust religious leaders (8.17), international (7.99) and local NGOs (6.58), health workers (7.73), and the Ministry of Health (7.43) more. The lowest levels of trust are for the opposition (4.07), people outside the community (3.73), and foreign people (3.24). Trust for the central government (5.70) and the President (6.36) are in a middle range. In terms of corruption, 82%, 77%, and 72% reported the police, the Tax Revenue Department, and the central government and legislature to be corrupt. More than half of the respondents also perceived the president, the local government, and the opposition to be corrupt.

### Empirical specification

We explore what characteristics predict an individual’s beliefs about the origin of Ebola at the beginning of the epidemic. First, we employ a Probit specification:

$$\mathrm{Pr}(Y_{i}=1)=\Phi (\alpha +S_{i}^{\prime }\beta +E_{i}^{\prime }\gamma +I_{i}^{\prime }\delta +D_{i}^{\prime }\phi +\varepsilon_{i})$$ (1)

where Pr(Yi = 1) represents our outcome of interest: the probability that respondent i was misinformed. **S**i, **E**i, and **I**i are vectors that include socio-demographic, economic, and information access variables for individual i, respectively. **D**i refers to a vector of self- reported measures of distrust toward different institutions (central and local government, the Ministry of Health, traditional and religious leaders, health workers, NGOs, family, and foreigners).

Second, we use supervised machine learning models–primarily Random Forests (RF) [42] and Least Absolute Shrinkage and Selection Operator (LASSO) estimation [43] to assess the relative importance of each of these characteristics in predicting who is misinformed.

There exist several machine learning methods, and determining which algorithm is best to use depends on many factors including the size, quality and nature of the data, the accuracy and interpretability of the output, the available computational time, ease of use and what exactly is the ultimate purpose. Since we wanted to quickly generate a numeric prediction in order to measure, in simple terms, the importance of predictors of the misinformed, we choose two algorithms that are easy to implement and can obtain results quickly, relying on decision trees and linear regressions.

Specifically, RF is a supervised learning method, based on decision trees where each one of them is a sequence of rules that divides the sample into groups based on certain variable cutoffs. The algorithm samples a random subset of the data and a random subset of the predictors to generate a prediction. The process is repeated multiple times (our estimates use 100 iterations), and the output is the average of the predictions among all trees. The LASSO is a regression analysis method that adds a penalization term based on the sum of the absolute values of the coefficients. By shrinking the parameters towards zero, it enhances the prediction accuracy and the interpretability of the model. The output entails only those variables that are the most relevant predictors.

Each model has weaknesses and strengths. LASSO is a simpler method and less prone to over-fitting, but it is incapable to identify complex relationships between the predictors and the outcome. While RF can handle well high dimensionality, over-fitting easily occurs and interpretability is harder. Both methods, however, measure well the importance of predictors of the misinformed: in RF importance is measured on a scale from 0 to 1 as the information gain is achieved when splitting on each variable. In the LASSO model the importance is determined by the estimated coefficients of the regression, where larger parameters (in absolute value) correspond to higher importance.

### Ethics statement

We obtained ethical approval from Duke University (C0910), institution of the Principal Investigator (EM) at the time of the study, and from the University of Liberia Pacific Institute for Research and Evaluation Institutional Review Board (UL-PIRE IRB) to access some of the proprietary data. Informed verbal consent was obtained for all the survey respondents. Only adults (18+) were interviewed.

## Results

### Predictors of misinformed

**Fig 1:**
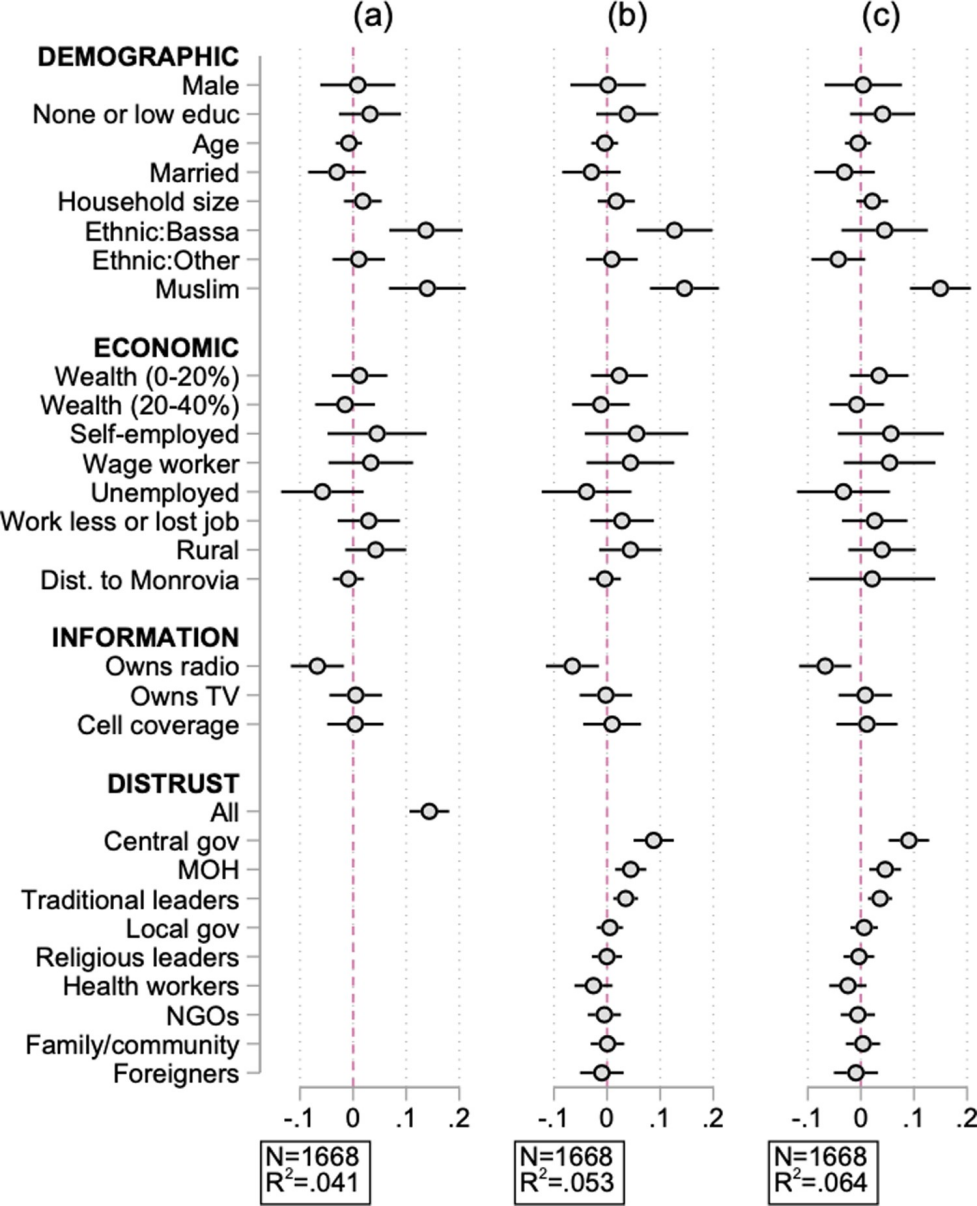
Profile of misinformed individuals. Dots present marginal effects from Eq 1. Spikes give 95% confidence intervals. Wealth index is constructed following the Demographic Health Surveys wealth index through a Principal Component Analysis. “Work less (2013)” equals 1 if individual worked less or lost the job since the beginning of the epidemic (in December 2013). The Distrust index is created using the −1 × (z − score) obtained from the normalization of the reported trust for a given institution on a 1–10 scale with 10 being highest trust. “Central gov” refers to national governmental institutions: the central government, the president, the legislature. “Foreigners” refers to people outside the individual’s community or foreigners. The excluded category for Muslim is Christian and other/no religion, which accounts for less than 4% of sample. Kpelle is the comparison category for ethnic groups. Kpelle is the majority ethnic group in Liberia. Panel (c) includes county fixed-effects.

Fig 1 presents the marginal effects obtained from the Probit model (Eq (1) in Methods and Material) which uses several socio-demographic, economic, and information access characteristics along with self-reported measures of institutional trust to predict the likelihood that an individual is misinformed. We define the distrust index as −1×(z−score) where the z−score is obtained from the normalization of the ordinal 0 to 10 level of trust reported. Panel (a) uses the average of all z-scores to create a measure of overall distrust. Panel (b) separates the distrust measure by type of institution. Panel (c) replicates the results in panel (b) but adding county fixed effects.

We find that demographic and economic characteristics are poor predictors of whether individuals are misinformed (Fig 1 panel (a)): misinformed individuals are not any less educated, older, poorer, more rural, or more economically distressed than their informed peers. For the most part, they do not have less access to sources of information: TV ownership and cellphone coverage is similar among informed and misinformed individuals. However, consistent differences arise along two dimensions: misinformed individuals are significantly more likely to report high levels of distrust, and less likely to own a radio. Specifically, a one standard deviation increase in the distrust index increases the likelihood that an individual is misinformed by about 14 percentage points (p = 0.000). On the other hand, individuals with radio access are 7 percentage points less likely to be misinformed (p = 0.008).

When we split the distrust measure by institution type (Fig 1 panel (b)), we find that the predictive power of the distrust measure is driven mostly by distrust in the central government (president, legislature, and national government), the Ministry of Health, and traditional leaders. Misinformed individuals are very similar to their informed peers in levels of distrust towards the local government, religious leaders, health workers, NGOs, family, their communities, and foreigners. Although misinformed individuals are more likely to be distrustful of the Ministry of Health, they are not any more distrustful of health workers than informed individuals, suggesting that the result on trust towards the Ministry of Health is driven by general distrust towards central government institutions (of which the Ministry of Health is part of) rather than distrust towards health workers.

**Fig 2:**
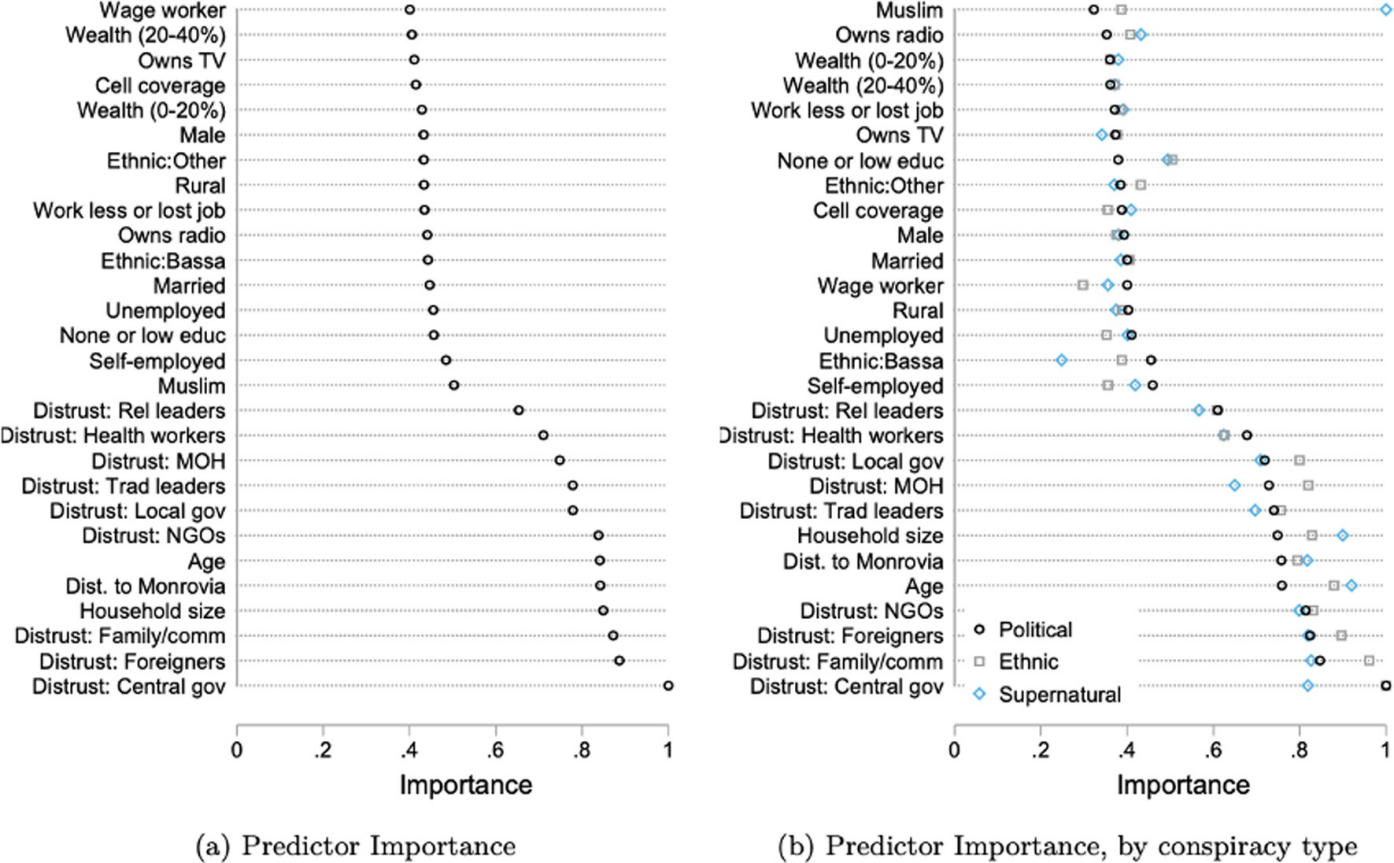
Random Forests (RF) predictor importance. Dots represent the importance score (from 0 to 1) of the predictors for being misinformed from Random Forests. Panel (b) presents the determinants of misinformation by type. “Political” refers to individuals reporting the government was responsible for bringing Ebola “Ethnic” refers to individuals reporting foreign NGOs, UNMIL, White, Fula, Kissi, or Mandingo people bringing Ebola. “Supernatural” refers to individuals reporting that God or witches were responsible for bringing Ebola. Refer to Fig 1 notes for a description of the variables.

This key finding about distrust for central governmental institutions is also evident in our machine learning results. When using RF, distrust in central governmental institutions is the predictor with a maximum score of 1, while socio-demographic and economic characteristics play a smaller role in predictive importance (Fig 2 panel (a)). This is further corroborated by the LASSO estimation, where only distrust in governmental institutions is reported as being an important predictor (coefficient LASSO: 0.042, Post-LASSO: 0.116). Instead, unlike the Probit estimates, the machine learning results do not support the conclusion that radio ownership is a key predictor of the misinformed.

We note additional findings. First, although the results in Fig 1 suggest that misinformed individuals are more likely to be from the Bassa ethnic group, this result disappears once we add county fixed effects. This points to a non-ethnic explanation for this correlation, therefore we consider ethnicity to be a poor predictor of the misinformed. This is further corroborated in the machine learning results (Fig 2 panel (a)), where the Bassa ethnicity does not appear as a key predictor. Second, we find evidence that Muslims are significantly more likely to be misinformed (see discussion next).

### Predictors of misinformed, by type of beliefs

**Fig 3:**
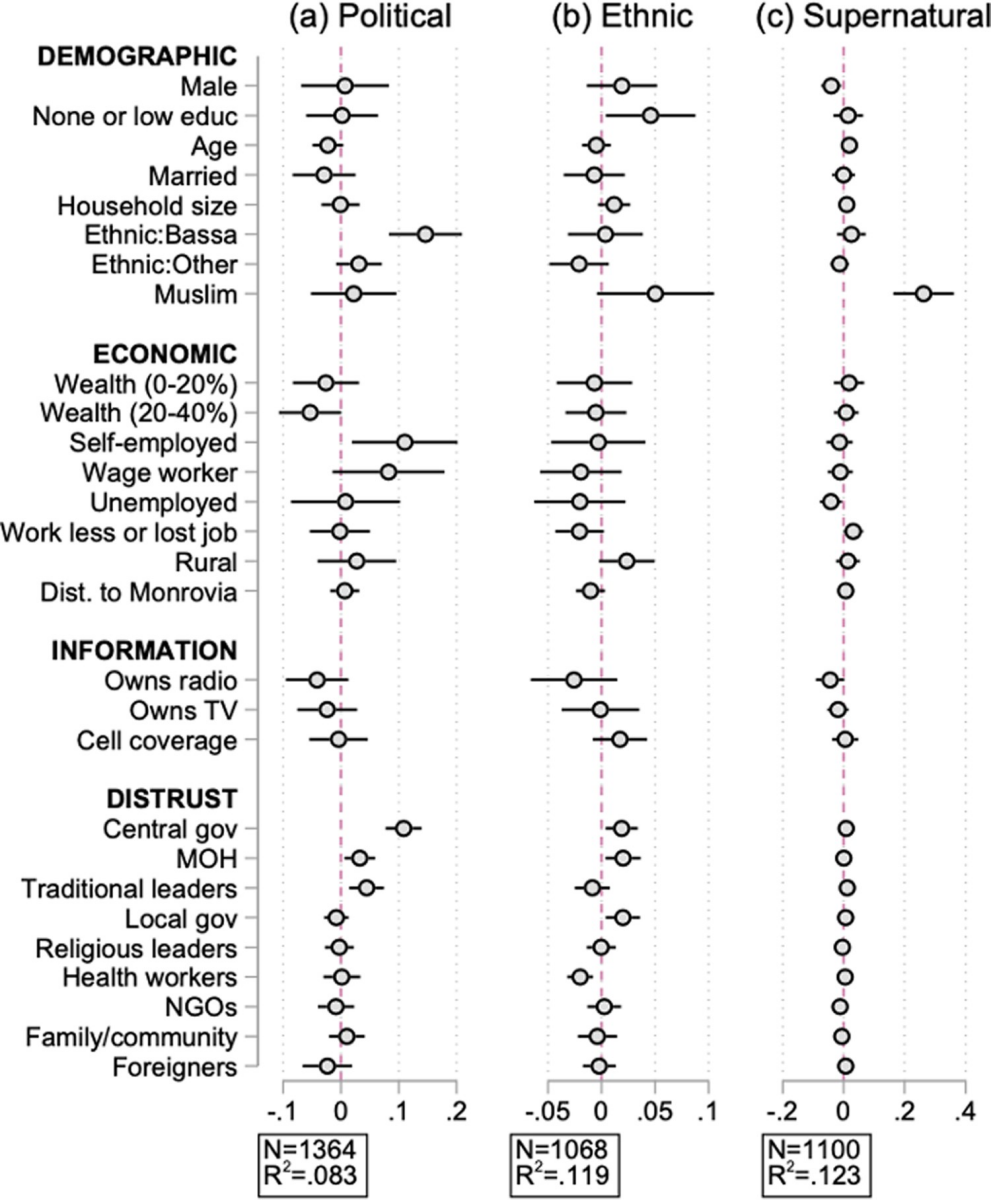
Determinants of the misinformed, by type of beliefs. Dots represent the marginal effects from Eq 1. Spikes give 95% confidence intervals. “Political” refers to individuals reporting the government was responsible for bringing Ebola to Liberia. “Ethnic” refers to individuals reporting foreign NGOs, UNMIL, White, Fula, Kissi, or Mandingo people bringing Ebola. “Supernatural” refers to individuals reporting that God or witches were responsible for bringing Ebola. Refer to Fig 1 notes for a description of the variables.

Fig 3 expands on the results presented in Fig 1, by considering specific types of beliefs. Panel (a) defines the outcome as equal to one if the respondent believes the epidemic has a political origin (i.e., individuals reporting the government being responsible for bringing Ebola to Liberia). Panel (b) looks at beliefs claiming an ethnic origin (i.e., individuals reporting White, Fula, Kissi, or Mandingo people, or foreign NGOs bringing Ebola to Liberia). Panel (c) looks at beliefs claiming a supernatural origin (i.e., individuals reporting God or witches being responsible for bringing Ebola to Liberia). Compared to the number of individuals in the sample who are informed about the origin of the epidemic (N = 997), about 27% of individuals believe a political origin story (N = 367), about 7% believe an ethnic origin story (N = 71), and about 9% believe a supernatural origin (N = 103).

We uncover several findings. First, distrust in the central government is the primary predictor of individuals assigning a political origin to the epidemic. A one standard deviation increase in the central government distrust index increases the likelihood that an individual believes a political origin by more than 10 percentage points (p = 0.000) and the machine learning results confirm this (Fig 2 panel (b)). As before, although Bassa ethnicity is significant in the Probit analysis, it scores poorly in the machine learning exercise and it becomes insignificant after including county fixed effects (not shown).

Second, the main predictors of ethnic origin beliefs are less clear. In the Probit model (Fig 3 panel (b)), distrust in the central government is a key predictor, although it is of lower magnitude than for political origin beliefs (panel (a)), and this is further confirmed in the machine learning results (Fig 2 panel (b)). One interesting result is that low education is a key predictor of ethnic origin beliefs, i.e., individuals with primary education or less are about 5 percentage points more likely to believe ethnic conspiracies (p = 0.009), but it is mostly irrelevant in other types. Again, the machine learning results confirm that educational level has a markedly higher importance for ethnic conspiracies relative to other types of beliefs.

Third, we uncover that the importance of Muslim religion predictor (Fig 1) is entirely driven by beliefs of supernatural origin. In fact, being a Muslim is trivial in predicting political and ethnic origin beliefs (Fig 3 panels (a) and (b)), but it is the single most important predictor of whether the individual assigns a supernatural origin to the epidemic (Fig 3, panel (c)). This is strongly supported by the machine learning results (Fig 2) in which Muslim religion is the highest score predictor. In Liberia, the Muslim population belong to the Mandingo and Fula ethnic groups, residing throughout the country, while those belonging to the Vai ethnic group live predominantly in the North-West, at the border with Sierra Leone and Guinea where the Ebola epidemic originated. In this part of the country, traditional practitioners include the secret Sande and Poro societies, seen both as religious and cultural practitioners which remain highly influential. In addition, the form of Islam practiced in Liberia is infused with traditional beliefs and oriented towards Sufism.

### Robustness

One concern with the distrust index in the analysis is that levels of (dis)trust were asked about six months post-epidemic and thus they can be affected by respondents’ exposure to the epidemic and the government’s response. By resorting to a large literature documenting a significant association between political distrust and preferences for political parties [44], we use voting preferences against the incumbent and major party of Liberian politics, the Unity Party (UP), during the 2011 election–three years prior to the epidemic–as a proxy for pre-outbreak distrust in the government. We also restrict the sample to individuals who reported voting for the same party in both the 2011 (pre-outbreak) and 2014 (post-outbreak exposure) elections, and thus we consider unchanged voting preferences as a likely proxy for unchanged government trust. We find that distrust in the central government, the Ministry of Health, and traditional leaders are still key predictors of the misinformed (not shown). We also show that individuals with low levels of trust for the incumbent party pre-outbreak (i.e., voted for the main opposition party in 2011, the Congress for Democratic Change, CDC) are significantly more likely to be misinformed (not shown). Finally, results are robust to using reported beliefs about corruption as an alternative measure of trust (not shown).

## Discussion

This article combines data on beliefs about the origin of the 2014 Ebola outbreak in Liberia with machine learning methods to predict who is more likely to be misinformed. By investigating a wide set of socio-demographic, economic and political factors associated with misinformation, we find that misinformed individuals are not any poorer, older, less educated, more economically distressed, more rural, or ethnically different than individuals who are correctly informed. More importantly, we show that trust in the government plays an important role in predicting who is more likely to believe misinformation. We also distinguish between types of beliefs by exploring whether respondents assign a political, religious or supernatural origin to the epidemic. We find that distrust in the central government is the primary predictor of individuals assigning a political origin to the epidemic, while Muslim religion is the most important predictor of whether the individual assigns a supernatural origin, and educational level has a markedly higher importance for ethnic beliefs.

As demonstrated by the recent COVID-19 pandemic, infectious disease outbreaks remain a worldwide threat [45]. Thus, the ability to identify misinformed individuals during epidemics remains a first-order policy issue. This may help establish better responses, allowing for more efficient targeting of information dissemination, improving individuals’ adherence to policies, and thus limiting the health and economic impacts of epidemics.

### Policy and research implications

Our findings have significant policy and research implications.

Distrust in the government, which is prevalent in countries such as Liberia, creates significant barriers to controlling disease and reduces healthcare utilization [4, 31]. Policies should address this root cause during normal times to limit additional costs during emergencies. In addition, information campaigns can be designed to address the profile of individuals characterized in this paper. Policy makers should carefully think about the role of state institutions in delivering health information. The typical strategy of a nationwide campaign with messages delivered by head of states or other governmental institutions could be ineffective when distrust towards those institutions is the single most important predictor of who believes the message. Indeed, contrary to expectations, it might be beneficial for political leaders to step back during times of crisis and let non-state agents carry the messaging.

Further research should be conducted to understand which are the most trusted sources of information and how the information content should be framed and disseminated, to maximize the effectiveness of communication strategies. While several institutions are trying to address misinformation [19], tech and public health experts need to be more proactive in educating populations, using contextualized approaches. It remains fundamental to help the public understand the adverse effects of misinformation and to validate information from trusted sources [46]. It would also be important to test to what extent messages delivered or endorsed by state versus non state institutions affect those believing in misinformation and their compliance with preventive behavior.

### Strengths and limitations

While past research has focused on quantifying misinformation and its effects during the 2014 West Africa Ebola epidemic or during Covid-19 [7, 14], this paper advances our knowledge of *who* are the misinformed. It contributes to the limited quantitative evidence that investigates factors associated with misinformation, especially in developing countries. More importantly, it uses novel data gathered during an epidemic and rigorous empirical methods, to show that government trust is the most important predictor of the misinformed. There is more and more evidence from previous [4, 31] and current epidemics [47, 48], that trust in the government is an important factor determining intentions and health behavior, and, more specifically, limiting desirable prevention behavior during epidemics. The key take-away of this study is that trust in the government should be taken into account and explored as one important predictor of health misinformation and to make policies more effective and impactful.

We acknowledge a number of limitations to this study. First, the setting considered entails a specific low-income country with weak institutions and a history of low-trust in the government. Therefore, the generalizability of the results beyond Liberia might be limited due to cultural and political nuances, as political distrust may be different in scope and origin. Yet, we argue that the main learning lessons (how trust in the government matters compared to other socio-demographic and economic factors) can be drawn for countries with similar characteristics and/or for misinformation during health epidemics.

Second, the results represent associations rather than a causal relationship. However, we attempted to control for as many confounding variables as possible, without over-fitting the empirical model and falling into multi-collinearity problems. Yet, there are still valid concerns about other unobserved factors potentially influencing the relationship, e.g., among others, time and risk preferences and altruism. Larger data collection efforts should try to measure these additional covariates.

Finally, the variables used in the analysis are gathered through mobile-phone data collection and thus self-reported, and there might be reporting or recall bias. We argue that most of the socio-demographic and economic covariates used in the analysis are less prone to these type of biases and there would not be incentive for the respondents to lie about their education, age, tribe, employment status, etc. We also did not use variables such as income that could be subject to recall bias. Instead, we developed a wealth index that relies on assets. In addition, as far as trust measures are concerned, our survey instrument was modeled based on internally validated and extensively used tools such as the Afrobarometer surveys, conducted also in Liberia.

Despite these limitations, this study may be of particular interest to policymakers and governments who aim to address health misinformation during epidemics. Stakeholders should think about ways to address low levels of government trust as an important determinant of the effectiveness and impacts of their policies.

## Supporting information

S1 Data This include data for analysis. (DTA)
